# “A Woman Is a Puppet.” Women’s Disempowerment and Prenatal Anxiety in Pakistan: A Qualitative Study of Sources, Mitigators, and Coping Strategies for Anxiety in Pregnancy

**DOI:** 10.3390/ijerph17144926

**Published:** 2020-07-08

**Authors:** Armaan A Rowther, Asiya K Kazi, Huma Nazir, Maria Atiq, Najia Atif, Nida Rauf, Abid Malik, Pamela J Surkan

**Affiliations:** 1 Department of International Health, Johns Hopkins Bloomberg School of Public Health 615 N. Wolfe St., Baltimore, MD 21205, USA; akazi5@alumni.jh.edu (A.K.K.); psurkan@jhu.edu (P.J.S.); 2 Human Development Research Foundation House No 06, Street No 55, F-7/4, Islamabad 44000, Pakistan; huma.nazir@hdrfoundation.org (H.N.); maria.atiq@hdrfoundation.org (M.A.); najia.atif@hdrfoundation.org (N.A.); nida@hdrfoundation.org (N.R.); abid.malik@hdrfoundation.org (A.M.)

**Keywords:** prenatal anxiety, women’s empowerment, mental health, anxiety, pregnancy, South Asia

## Abstract

Common mental disorders are highly prevalent among pregnant women in low- and middle-income countries, yet prenatal anxiety remains poorly understood, particularly in the sociocultural context of South Asia. Our study explored sources, mitigators, and coping strategies for anxiety among symptomatic pregnant women in Pakistan, particularly in relation to autonomy in decision-making and social support. We interviewed 19 pregnant married women aged 18–37 years recruited from 2017–2018 at a public hospital in Rawalpindi who screened positive for anxiety. Thematic analysis was based on both inductive emergent codes and deductive a priori constructs of pregnancy-related empowerment. Gender norms emerged as an important dimension of Pakistani women’s social environment in both constraining pregnancy-related agency and contributing to prenatal anxiety. Women’s avenues of self-advocacy were largely limited to indirect means such as appeals to the husband for intercession or return to her natal home. The levels of autonomy during pregnancy depended on the area of decision-making, and peer/family support was a critical protective factor and enabling resource for maternal mental health. Women’s disempowerment is a key contextual factor in the sociocultural experience of prenatal maternal anxiety in South Asia, and further examination of the intersections between empowerment and perinatal mental illness might help inform the development of more context-specific preventive approaches.

## 1. Introduction

With available data indicating the high prevalence of common mental disorders (CMDs) among pregnant women in low- and middle-income countries (LMICs) [1], maternal depression has remained a key focus of research and healthcare, while prenatal maternal anxiety in LMICs remains poorly understood [2,3]. Anxiety in pregnancy is a significant risk factor for poor maternal and child outcomes, including postnatal depression and preterm birth [4,5,6,7], and of particular concern in LMIC contexts like Pakistan where access to mental health services is scarce and the prevalence of preterm births is persistently high [8,9,10]. Efforts to produce a richer understanding of prenatal anxiety to inform the development of more context-specific approaches, however, are severely lacking [11].

In particular, there exists a paucity of qualitative studies exploring the complex experience of anxiety during pregnancy for women living in South Asia, where gender inequality and other barriers to maternal decision-making are important dimensions of prenatal anxiety [3]. This often includes the inability of women to make health-related decisions due to the control of husbands or in-laws over reproductive health and family planning [12]. Furthermore, prior research suggests that anxiety during pregnancy in Pakistan is not only highly prevalent (as high as 35–49%) but also closely related to low social support; lack of support may serve as both a risk factor for prenatal anxiety and mediator of its relationship to other risk factors such as female gender of previous children [13,14].

Given that much of the literature on prenatal anxiety is derived from English-speaking high-income settings [15,16,17], current understandings may fail to capture features particular to other sociocultural contexts, especially as they relate to family and social support [3]. Moreover, previous efforts to conceptualize or measure psychosocial stressors in South Asia have either failed to include women’s control over pregnancy-related decision-making or represent this dimension solely in terms of financial autonomy or family planning [12,18,19]. Alternatively, autonomy in decision-making and level of social support during pregnancy may be more holistically represented as constructs related to the multidimensional theory of empowerment [20].

Under a processual understanding, women’s empowerment refers to their acquisition of power to make choices, which in turn consists of having agency to make strategic life decisions using available enabling resources (human, material, and social) to attain achievements (such as improved mental or physical health) despite contexts of constraint [21,22]. For childbearing women, included within the scope of these three interrelated and inseparable dimensions of power is having the voice and ability to make skillful decisions for improving their pregnancy-related health (in this case, improved coping or reduced severity of psychological distress such as from prenatal anxiety), as enhanced or constrained by levels of social support and relationships with family, peers, and healthcare providers while pregnant [20]. This may be thought of in terms of pregnancy-related empowerment constructs [23]: provider connectedness, or an antenatal care relationship that minimizes power differentials between patient and provider; skillful decision-making, or the process by which women evaluate and choose a direction relevant to their health; peer connectedness, or the bond between women that develops from caring and supportive relationships; and finally, gaining voice, or the ability of women to be knowledgeable about their health and advocate for health care options.

**Figure 1:**
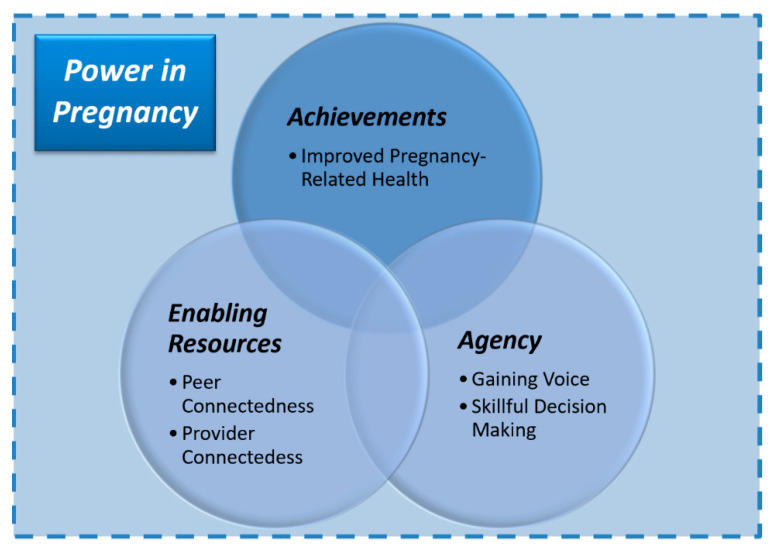
Women’s empowerment, sources of anxiety, and coping strategies in pregnancy: conceptual framework adapted from Kabeer’s (1991) model for women’s empowerment and Klima et al. (2015) constructs for pregnancy-related empowerment to understand the sociocultural experience of maternal prenatal anxiety as it relates to maternal decision-making and social support in the South Asian context.

Informed by these constructs as parts of an adapted conceptualization of power in pregnancy (Figure 1), the aim of our analysis was to explore sources, mitigators, and coping strategies for anxiety during pregnancy from the perspective of symptomatic Pakistani women and thereby further our theoretical understanding of the sociocultural experience of prenatal maternal anxiety in South Asia, particularly in relation to women’s autonomy in decision-making and level of social support from an empowerment perspective.

The corollary concept of disempowerment implies a condition of denied choice [22], which may hold immense relevance to investigating the phenomenon of prenatal anxiety within the cultural context of South Asia. Although diverse forms of *purdah* (the separation of women from unrelated adult men) are observed across the subcontinent, gender norms linked to *purdah* as a social institution are central to regulating women’s autonomy before and after marriage in Pakistan, particularly with respect to restricting public visibility and mobility [24,25]. Beyond traditional *purdah* observances, cultural practices like patrilocally extended households (in which a married woman lives with or near her husbands’ parents [26]) shape and constrain agency for pregnant women throughout South Asia, where such patriarchal patterns cut across religions and regions [27,28].

While gender roles have been posited as potentially contributing to gender gaps in mental health, with women experiencing significantly higher rates of anxiety and mood disorders worldwide [29,30], prior studies on the role of women’s disempowerment in anxiety remain sparse [21], with no such prior studies conducted in South Asia. Our study aims to begin addressing this gap in the literature through a qualitative exploration of autonomy and anxiety during the critical and potentially vulnerable period of pregnancy in Pakistan.

## 2. Materials and Methods 

This qualitative research was a secondary in-depth analysis of interviews with expectant mothers conducted from September 2017–August 2018 in Rawalpindi District, Pakistan, as part of a larger study investigating the cultural context of perinatal anxiety to inform the development and testing of a prenatal prevention intervention delivered by female non-specialist providers focusing on anxiety in pregnant women [31]. Pakistan is a middle-income country with persistently high rates of maternal and infant mortality [32], and Rawalpindi District is located in the northernmost region of the Punjab province adjacent to the nation’s capital of Islamabad. Rawalpindi includes a mixture of urban, periurban, and rural populations typical of a low-income setting of an LMIC with high rates of poverty (29.5% below poverty line), high fertility rates (3.8 live births per 1000 women), low female literacy rates (45%), and large average household sizes (6.2 persons) that can include extended and joint families [33]. Participants were recruited from among patients at the Obstetrics and Gynecology Department of Holy Family Hospital (HFH), a large public tertiary care facility at Rawalpindi Medical University that provides free or low-cost antenatal care to a catchment population of over 7 million. Ethical approval for this research was obtained from the Institutional Review Board (IRB) of the Human Development Research Foundation in Gujar Khan, Pakistan, and the IRB of the Johns Hopkins University Bloomberg School of Public Health (IRB code 7678).

We conducted semi-structured in-depth interviews covering sources, manifestations, and coping strategies for anxiety during pregnancy as well as common psychosocial stressors with 19 symptomatic pregnant women attending the outpatient department of HFH (Table 1). Trained research assistants approached consecutive pregnant women attending the clinic and sought consent to participate. The participants were screened for symptoms of anxiety using the Urdu adaptation of the Hospital Anxiety and Depression Scale (HADS) [34,35,36], which has shown good reliability and validity in the Pakistani context [37], and were administered the Structured Clinical Interview for DSM-5 (SCID) [38]. The SCID is a semi-structured interview guide for major DSM diagnoses administered by a clinician or trained mental health professional to patients (psychiatric or general medical) or individuals who do not identify themselves as patients. Women who screened positive for being at least “at risk” of anxiety according to the HADS (i.e., cutoff ≥ 8 on the HADS anxiety subscale) in the absence of clinical depression or other serious medical conditions, resided within 20 km of Holy Family Hospital, and understood Urdu were eligible for inclusion in the study. The exclusion criteria included the diagnosis of a major depressive episode per the SCID, severe depression or suicidal ideation, and self-report of past or current other psychiatric disorders (e.g., bipolar disorder, schizophrenia) or psychiatric care (e.g., the use of anxiolytic medications or other psychotropic drugs). Recent psychometric approaches suggest an underlying unidimensionality for all 14 items of the HADS, for example, as a global measure of psychological distress [39]. However, our conceptual focus on the clinical or subclinical symptoms of prenatal anxiety (not otherwise attributable to a physical cause or coexisting general medical condition [40]), most notably pregnancy, informed our use of the seven-item anxiety subscale only (Table 1).

**Figure 2:**
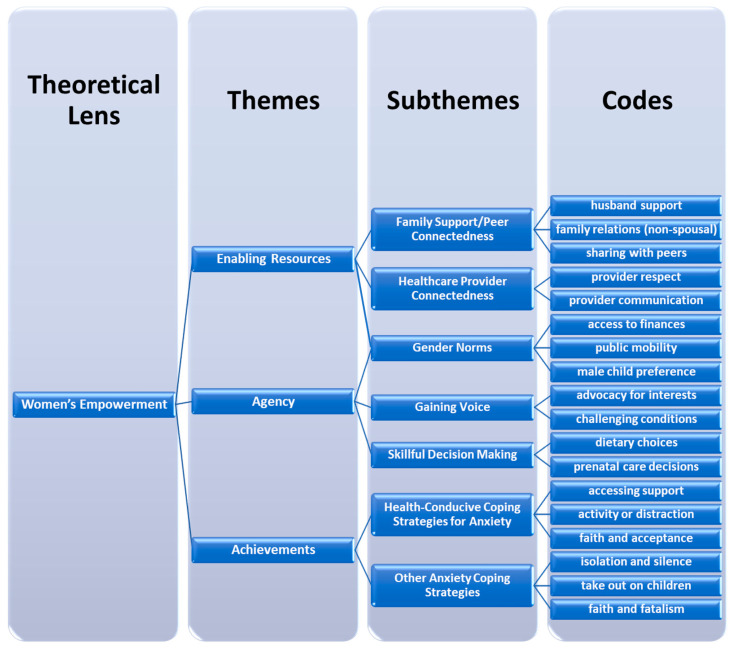
Schematized final codebook combining inductive, emergent codes with deductive, a priori themes from conceptual framework of pregnancy-related power.

The interviews were recorded and transcribed verbatim in Urdu prior to translation and analysis. We analyzed the interviews thematically using both emergent codes for common social or cultural factors found to be contributing to stress in pregnancy, manifestations of anxiety, and coping strategies employed by pregnant women, as well as coding for relevant a priori constructs of pregnancy-related empowerment [23]. First, the emergent codes and categories were derived independently by two bilingual members of the research team (AAR and AKK) through the open coding of a subset of four initial transcripts [41,42], followed by discussion and the development of a common thematic framework. Then, the resulting codebook was refined for the aggregation of emergent codes under themes and subthemes, which included both inductively derived categories and deductively applied constructs from an adapted conceptual framework of pregnancy-related empowerment (Figure 2) [22,23]. This final codebook was applied to all the transcripts using the Dedoose software (Dedoose, Version 8.2.14, Los Angeles, CA, USA). Analytic memos by coders, frequent revisiting of the raw data in Urdu, and periodic meetings to oversee the reconciliation of coding discrepancies and refining of subthemes were used to generate the final thematic analysis and key results.

## 3. Results

All 19 participants were pregnant married women (Table 2), nearly half of whom were primigravida, and all the interviews took place over 45 to 60 min in a private area of the HFH. The mean age of the participants was 26 years (range 18–37 years) and the mean length of schooling was 10 years, with the sample ranging from no secondary education to 16 years of schooling. Of the 19 women, 13 lived in a patrilocal residence or “joint family” arrangements with their *saas* (husband’s mother), *susar* (husband’s father) and/or other members of their *susral* (husbands’ family). In the following results, all the participants are referred to by researcher-assigned pseudonyms.

The emergent codes and findings with respect to the sources and mitigators of anxiety reported by the pregnant women were divided into five sub-themes, of which two were considered to be connected to agency as a dimension of women’s empowerment (Gaining Voice, Skillful Decision-Making), two were part of women’s enabling resources (Family Support and Peer Connectedness, Healthcare Provider Connectedness), and one was related to both enabling resources and agency (Gender Norms). Of the six strategies for coping with anxiety that emerged from the women’s accounts, three were classified as health-conducive, and all were viewed from the perspective of mental health-related achievements in the empowerment framework.

### 3.1. Agency

#### 3.1.1. Gaining Voice

Among the pregnancy-related empowerment constructs that applied to women’s accounts of their anxiety was that of gaining voice, or the perceived ability to be knowledgeable about their health and advocate for themselves or their families. Many women with symptoms of anxiety, particularly among those who lived in joint families, expressed having little to no ability to challenge or change their circumstances in the context of marriage and domestic duties. Khadija, a 24-year-old woman with only five years of formal schooling who was in her seventh pregnancy, summarized her sentiments as follows: “A woman is a puppet; she only knows to cook the meals and wash the clothes of her husband and to care after the children” (M10). The perceived barriers to exercising greater voice or self-advocacy (especially in relation to the burden of household duties and to reproductive health decisions) included fears of causing arguments with one’s husband, upsetting one’s mother-in-law or other family, or going against the will of God and thus worsening their condition. Asked to elaborate on the sources of her fears during her pregnancy, 18-year-old Chaandni said, “I feel something bad could happen like an argument with my husband” (M8), the possibility of which she attributed to her either falling short in the performance of chores or raising the topic of finances and thereby provoking anger. These barriers were frequently described among those women who also expressed hesitance, fear, or lack of choice for exercising voice in reproductive health decisions, including family planning or, in two cases, the elective abortion of unwanted pregnancy. Zara, a 20-year-old mother of two with no secondary education, stated, “I think of aborting but I’m scared of going against *Allah*’s (God’s) will and how it may affect my children” (M14). Zara then proceeded to tell the interviewer how, as a consequence of a prior abortion, her elder child had fallen ill.

Among the minority of women who described higher levels of voice at home, their means of self-advocacy were primarily indirect, consisting largely of either appealing to one’s husband for intercession or leaving *sasural* (marital) homes temporarily to return to their *maike* (natal) homes. Raabia, a 37-year-old woman who felt overburdened by chores and neglected by her in-laws during periods of her husband’s absence (who worked overseas), described once asking her husband, “‘Please do one thing for me and leave me at my parent’s home, where I have honor’ (…) I became very anxious about my situation” (M13). Reliance on seeking a husband’s support or leaving for a natal home was observed not only among women who felt only mistreated, like Raabia, but also in more extreme cases such as that of Zahirah, a 34-year-old mother of four who felt pressured to conditionally promise the custody of her unborn child to her husband’s childless sister: “My *nand* (husband’s sister) doesn’t have children, and she said that if I have a baby girl, then she will adopt her (…) I asked my husband to support me in this situation as I am anxious about it; they might create problems after my delivery” (M18). Zahirah described having delayed her first pregnancy (with her husband’s permission) until feeling ready three years into her marriage, yet she felt that the demands of household responsibilities had kept her from the joy of watching her previous children grow up and thus wanted another child after many years: “My husband didn’t want this pregnancy, but I insisted on this baby” (M18). Despite demonstrating among the highest levels of pregnancy-related voice and agency of our study participants through these prior decisions, Zahirah still conveyed uncertainty and dependence on her husband’s intervention to express and exercise refusal of her sister-in-law’s request to adopt her child. Thus, for many women, fears about the potential consequences of exercising voice or self-advocacy in relation to their health or pregnancy functioned as both sources of anxiety in and of themselves as well as barriers to addressing the circumstances contributing to their experience of prenatal anxiety.

#### 3.1.2. Skillful Decision-Making

A closely related empowerment construct that appeared throughout the data was skillful decision-making, or the ability to evaluate and make choices that would impact their health [23], namely regarding diet or healthcare utilization. Diet-related decision-making represented an area of relative freedom and control for pregnant women, perhaps due to most remaining responsible for meal preparation for the entire family: “Mostly they (pregnant women) are worried about what to cook and what their family members will like to eat” (M8). In a few of the women’s accounts of their prenatal anxiety, the consumption of certain foods served as an explanation for physical symptoms, a remedy for emotional distress, or a decision made with advice but not requiring permission from others. Saaliha, a 27-year-old woman with 16 years of schooling (among the highest levels of formal education among participants) and a job tutoring, recounted one instance of such dietary decision-making: “When I feel anxiety, I want to eat something (…) I usually eat what I like the most, as I have all the things to eat in my kitchen. But yesterday, I wanted to eat homemade *dahi baray* (fried flower balls in yogurt) but I did not have yogurt at the time, so I left my chores and waited for one of my students. I had my student bring yogurt for me, made *dahi baray*, and then I felt relaxed” (M15).

In contrast, healthcare decision-making, especially for accessing prenatal care, often required the permission of one’s husband or in-laws. In some interviews, this was stated as a given fact or linked to general restrictions against a woman traveling outside the home unaccompanied by elders or male family members, as in the case of 21-year-old Pashmina in her first pregnancy: “My family won’t allow me to go outside alone (…) actually, there is a bus station outside which is why women of our home are not allowed to go outside on a frequent basis” (M3). Others attributed this to the fact that, for a woman to visit a health facility, her husband would need to pay the associated expenses and her family would have to help manage other children or chores at home. Jumana, a 24-year-old mother of two who lives in a joint family, described multiple restrictions on her travel to the health facility, first stating, “It’s very difficult for me to come to the hospital (…) my kids are too young so I can’t leave them alone” and later describing her general mobility as follows: “I don’t visit my neighbors without their (husband and in-laws’) permission. I only visit my parent’s house; they don’t like me to go anywhere” (M7). Levels of women’s autonomy in pregnancy thus varied depending on the area of decision-making involved as well as the degree of family involvement and permission required, which in turn appeared to shape the role of these processes in their experiences of anxiety.

### 3.2. Enabling Resources

#### 3.2.1. Family Support and Peer Connectedness

The quality of a woman’s relationships within her household—primarily with her husband and his mother and sisters—emerged as critical enabling resources for empowerment and important factors in experiences of prenatal anxiety. Many women described their husbands as having the potential to substantially alleviate the symptoms of anxiety depending on the extent to which they responded with either consolation or dismissal of their expressed distress. Khadija, who had felt so little autonomy in decision-making as to describe women as “puppets”, went on to say, “If the husband will speak a few words of love to his wife, all her tensions are removed” (M10). In addition to providing verbal sympathy, forms of spousal support included listening to them talk about stressors, showing concern or offering some reassurance about their health, and buying things for them. Saaliha, who described her husband as an age-fellow with whom she had studied prior to marrying, was especially emphatic about her husband being a source of confidence and relief from anxiety: “He never scolded me, never said anything to me. I feel that nobody gives me as good advice as my husband (…) my husband has given me confidence, and he is my best friend” (M15). In contrast, Khadija described criticism from, conflict with, and unsupportive behavior by her husband as a major contributor to her anxiety: “There are more problems when my husband says something like, ‘You have not done this work at home, where were you all day?’ This makes me mentally distressed, and you can’t tolerate such things said by the person who cares about you the most” (M10). The husband was thus characterized by several women as someone intimately close whose emotional support or lack thereof could be especially potent as a mitigator or exacerbator of anxiety in pregnancy, respectively.

The importance of the spousal relationship was especially high for women who experienced frequent or severe conflict with their mother-in-law or sisters-in-law. When asked about the greatest source of worry and stress for herself and other women during pregnancy, Raabia described her situation as follows: “Mostly women face greater problems related to her in-laws. The biggest issue is that women are enemies of other women (…) A man works and leaves the home. At home, women are eating other women, women are killing other women. I think to myself, ‘why is she doing this?’” (M15). Such sentiments of vulnerability to the negative treatment of in-laws was not limited to those women living in joint families. Zahirah lives in a separate flat with her husband but nonetheless described her circumstances with respect to her in-laws as follows: “No one has helped me, they only talked behind my back (…) they tried to create more problems for me. However, what matters is the support and cooperation of my husband” (M18). Verbal criticism from or arguments with a mother-in-law were the most common forms of negative non-spousal interactions mentioned. A few women described maltreatment in the form of denying expectant mothers any practical assistance with her household chores, even during the later months of pregnancy, as was the case with Raabia: “I wash my child’s clothes myself, whether I have strength or not. I wash my own clothes (…) I do all my work myself, even making *roti* (flatbread), whether I have strength or not, whether I live or die” (M15). For other mothers, the absence of sympathy or emotional support, rather than practical help, was described as the primary source of anxiety. Hawa, a 24-year-old mother of two, said, “There should be family support; not a lot, not with household chores. They don’t have to do your work, but even if they say a word or two and they sympathize, then all your fatigue goes away” (M9).

While the majority of women described in-laws as contributing to their anxiety through poor treatment or lack of support, many gave additional or alternative accounts of mothers-in-law still being crucial pillars of support during pregnancy, particularly through providing health advice and accompanying women to prenatal appointments. Asked whether she had family assistance and support during her pregnancy, Chaandni reported, “If I had to visit my doctor, my mother-in-law went along with me” (M8). When Chaandni felt “bodily pains”, she discussed this with her mother-in-law, who promptly took her to a doctor, and Chaandni described her serving as an advocate in healthcare encounters. Teena, also in her first pregnancy, mentioned receiving practical assistance with housework: “My mother-in-law sometimes helps me in the kitchen (…) I just don’t feel good making *roti*, so my mother-in-law helps me” (M5).

A pregnancy-related empowerment construct that emerged as intimately tied to family support in several accounts of prenatal anxiety was peer connectedness, or bonds between women that develop from caring and supportive relationships [23]. Many women described feelings of loneliness or isolation, due in part to either the physical burden of pregnancy or the domestic obligations of married life limiting their ability to travel and visit with friends and family. Loneliness was described as both a source of anxiety as well as an exacerbator of anxiety symptoms, as explained by Hawa, whose own symptoms included feelings of restlessness, frequent arguments, and suspicions that others’ private conversations were about her. After describing the importance of sympathy from family, she later discussed her negative thinking as follows: “I think a lot of responsibility goes to family members in making you feel (negatively); if they behave a little good, I mean, a pregnant woman shouldn’t be thinking such things (…) because she will be distracted by all these people happily. The lonelier and more isolated you are, the more negative thoughts occur” (M9). Hawa, who reported feeling unsupported by non-spousal family throughout her interview, thus describes the benefits of having family around (e.g., dispelling feelings of isolation and negative thoughts) as conditional on the connectedness of a good relationship including caring and supportive behaviors, something she felt was lacking in her relationship with in-laws: “I did not receive any kind of support yet. I have done everything by myself until now” (M9). Chaandni, who also lives in a joint family, provided a contrasting example of an emotionally supportive bond when she described often sharing her fears and worries with her sister-in-law: “When I share with her, she supports me” (M10).

The bonds described as most instrumental in alleviating a woman’s anxiety were those with other women who were either recently or concurrently pregnant and could thus empathize with her experience. The most illustrative example of such peer connectedness was Deena, whose *devrani* (husband’s younger brother’s wife) and *jethani* (husband’s older brother’s wife) were also pregnant at the time of her pregnancy: “We all would share feelings and cooperate with each other. And when one had problems, we would guide her: if someone says she is suffering from burning, (another sister suggests) she had *desi ghee paratha* (flatbread layered with clarified butter) due to which she’s feeling burning (and should) drink milk early in the morning, from which she felt relief (…) We all share experiences with each other” (M11) With specific foods again serving as remedies for physical symptoms, dietary choices were empowered by the enabling resource of shared insights and advice among peers connected as expectant mothers. Deena also went on to describe the benefit of having someone to provide reassurance about certain physical symptoms being normal or improving with time, thereby relieving her associated anxiety. Finally, such empathetic peers were a source of practical help: “When I was in my third month, I used to wash clothes. She (my sister-in-law) said no, and she started washing and ironing them when I was in my fourth month (…) we all would empathize with each other and all do a portion of the work” (M11). Such practical and emotional support afforded by peer connectedness among women, especially within households, emerged from women’s narratives as a potent mitigator of their anxieties.

Greater hesitation was expressed with respect to sharing experiences, especially fears or negative experiences, with peers outside the home. Deena, who had described in detail the strength of her bonds with her sisters-in-law, attributed her avoidance of expressing distress to friends or even health care providers to fear of embarrassment, judgement, or being looked down upon: “Some people will say that I’ve been hurt by my husband, so I don’t discuss it with anyone. I am perceived positively in the eyes of people, that I am very happy in my life, so I don’t want that image to be distorted. I don’t want to feel low in the eyes of other people” (M11). Deena thus draws a distinction in her level of comfort sharing her anxieties with those within versus outside the joint family household. Many other women refrained altogether from disclosing their distress to others, as discussed further in the context of isolation (See Other Coping Strategies). Zara, who lives separately from her in-laws, explicitly mentioned embarrassment as the reason for not sharing her anxiety symptoms with anyone outside of her husband: “I feel embarrassed (…) they will laugh” (M14).

#### 3.2.2. Healthcare Provider Connectedness

Another pregnancy-related empowerment construct that appeared in many mothers’ accounts as an influential factor in experiences of prenatal anxiety was the theme of provider connectedness, which describes the therapeutic relationship and to what extent it minimizes power differentials and creates an environment of trust and respect [23]. For many women, descriptions of the potential benefits of confiding in doctors contrasted sharply with accounts of their actual medical encounters for prenatal care, which were often depicted as either creating or exacerbating their feelings of anxiety. These experiences included being spoken to rudely or feeling insulted by doctors, being rushed through visits or bounced between different areas or providers of the clinic, and feeling as though care was denied or delayed arbitrarily. Saaliha recounted witnessing other patients being publicly scolded during her last visit to the hospital and said, “I fear that I will also be similarly disgraced in front of other mothers” (M15). Zara drew a direct connection between her avoidance of discussing childbirth-related fears with her doctor and the expected reaction from healthcare providers: “I feel the doctors will insult me” (M14). With respect to sharing psychosocial stressors or symptoms of anxiety with their providers, Khadija explained that such disclosures outside the household would be unlikely to be permitted by husbands or mothers-in-law who often accompany women to hospital visits: “If her mother-in-law and husband sit in front of her and you ask her life-related questions, she will not give you clear answers” (M10). Reluctance rooted in negative experiences or fears of the reaction from either healthcare providers or family members thus served as barriers for many women against utilizing the provider relationship as an avenue to share or address their anxieties in pregnancy.

The minority of patients who conveyed higher levels of provider connectedness focused their accounts on the potential for doctors who exhibit kindness and exercise empathetic listening to serve as confidants. When asked about the qualities in a provider that encourage greater communication and trust, Raabia emphasized love and understanding: “She should be someone who tries to understand the patient. The most important thing is to speak to the patient with love” (M13). Erum, a 24-year-old with 10 years of education, expressed similar sentiments: “The doctor should also be good and patient. She (the mother) should be able to talk to her doctor from her heart, like I did just now. Right now, my sister is not here with me; that’s why I have discussed my problems with you” (M1). Medical providers were thus viewed as potential mitigators of anxiety among pregnant women, but the negative personal and vicarious experiences of many led to apprehension or anxiety being associated with hospital visits. Such sentiments were a significant barrier against healthcare providers serving as an enabling resource for women to address sources of anxiety, especially those related to childbirth, physical symptoms, or general pregnancy concerns.

### 3.3. Gender Norms

Gender norms were an important factor interwoven throughout women’s accounts of anxiety during pregnancy, one that intersected with and spanned across both enabling resources and agency as empowerment domains. This includes manifestations of gender norms through not only previously discussed restrictions on women’s mobility, but also in matters related to household finances and child gender preferences. With respect to finances, the scope of women’s sources of anxiety extended beyond general financial problems to also include specific constraints they perceived in their ability to access their husband’s income or household assets. General financial concerns included costs related to pregnancy and additional children (“My husband’s earning used to be good enough, but now expenditures are increasing day by day.” M8), or the loss of a husband’s employment or income. This latter concern was the primary source of anxiety for Basma, a 28-year-old mother of three who, when asked whether she’d experienced any major changes since the start of pregnancy, said, “my husband’s job was terminated (so there is) constant tension about what will happen; there is no income, there is nothing. What will we do?” (M2). Even in the absence of economic insecurity for the household, several women expressed distress over what they viewed as the inequitable or inadequate allocation of the husband’s income for their or their children’s needs, especially in the context of joint families. Erum expressed frustration over what she viewed as disproportionate spending on her sister-in-law (“He does not give me any money but gives it to her whenever she comes.” M1), Deena felt stress related to her husband supporting the entire joint family (“My husband is supporting them because it’s a joint family system (…) this is my only cause for tension.” M11), and Raabia made reference to multiple disputes with her in-laws over the portion of her husband’s income spent on her and her child. Underlying her concerns were deeper insecurities about securing assets for the future welfare of her children, as revealed when she compared herself to her sister-in-law: “Because my husband has managed the home from the start, he supports his mother and father (…) what will happen with my child? My sister-in-law is not like me, she is very different. They bought two shops and a plot of land. My husband should think about our children” (M13). Paid employment was uncommon among women in our study, and their means of accessing household income as an enabling resource often centered on seeking influence over the financial decisions of their husbands or competing with the husbands’ family for access to his income.

Another common manifestation of gender norms that functioned as both a source of anxiety and influential factor in women’s autonomy was the strong preference for a male child. In some cases, the gender of the child was expressed as a general source of anxiety during pregnancy (“The worry during pregnancy is mostly thinking about whether it is a son (…) that is why women get anxious.” M10), with social pressure on women to give birth to more sons rather than daughters (“People said, ‘She has one son, so it will be good if *Allah* gives her another son.’” M2). Erum, the mother of one daughter, described much more direct expressions of male preference by her husband and in-laws, who had prepared for and expected a son and, disappointed by the birth of her daughter, immediately withdrew any emotional support: “Everything was bought for a son (…) Everything. So, when I was in hospital, they left me alone (…) my husband did not love her (the baby). Once he took her—to throw her away (…) I feel afraid now, like this. I think, what will happen if it is a girl again?” (M1). The fears of having another daughter and the consequences this would have in terms of her marriage and her child’s welfare were raised throughout Erum’s account of her experiences with prenatal anxiety. As explained by Zahirah, who was under pressure to let a childless sister-in-law adopt her child if she had another girl, “people think that daughters are a burden to parents” (M18). Thus, the perceptions of those outside the household as well as the behaviors of husbands and in-laws reinforced a strong preference for male children, for which the burden of responsibility was placed on expectant mothers and served as a major source of anxiety during pregnancy. Gender norms overall, including the control of household finances and expectations regarding bearing male offspring, were significant sources of prenatal anxiety that shaped and defined, in part, women’s access to enabling resources and exercise of agency during their pregnancies.

### 3.4. Achievements

#### 3.4.1. Health-Conducive Coping Strategies for Anxiety

Among the ways that pregnant women described trying to address or reduce their anxiety were four shared approaches that could be considered health-conducive from the perspective of women’s mental health as an achievement within the framework of pregnancy-related empowerment. For those women who had family support and peer connectedness available to them as enabling resources, accessing emotional and practical support was an important strategy for addressing anxieties, which several women described benefiting from substantially. For different women, accessing support took different forms, such as sharing one’s feelings, seeking reassurance or advice, or getting assistance with household chores or prenatal visits. Chaandni described employing multiple avenues of support: “My mother-in-law accompanies me to the hospital and to get my medications (…) I talk to my sister-in-law, sit down and talk to her (…) then my heart gets a little bit lighter” (M8). Aaliya, a 25-year-old also in her first pregnancy, sought emotional support in the form of reassurance from her own mother and sister rather than in-laws, stating, “I talk to my mother or sister, (telling them) that I don’t know what will happen. Then, they make me understand that there is no need to worry. My husband also makes me feel calm.” (M19). Basma went beyond the household to seek practical help from her neighbors: “My neighbors are very nice; whatever chores I have, they come and do those for me…(when) I tell all my neighbors that I am not feeling well today.” (M19).

Apart from accessing emotional or practical support, two other health-conducive strategies for coping with prenatal anxiety included seeking positive activities or distractions and employing faith-based forms of coping or acceptance. Zulekha described making herself busy with “different household tasks or in stitching clothes; stitching clothes is my hobby” (M12), Teena would ask her husband to take her outside where she could “go out in a cool breeze and enjoy the weather so I feel better” (R5), and Surayya used caring for her other children (“feeding my baby” M6) to take her mind off of things that worried her. Other mothers described seeking relief in religious practices such as the recitation of *Quran*, remembrance of God (*zikr*), saying of prayers (*namaz*), invoking God’s assistance (*dua*), or prostrating to God (*sajda*). One of these mothers, Deena, described the role of faith in her response to concerns about the health of her unborn child: “Sometimes I feel him making movements and think maybe there is some problem with him, then I start saying *Bismillah* (in God’s name) spontaneously, and I pray for his safety.” Among women whose accounts reflected relatively greater enabling resources or agency in pregnancy-related decisions, faith-based practices and reliance on God’s will were described as providing peace of mind: “I just have firm belief in *Allah*, that whatever will happen will be good for me” (M8).

#### 3.4.2. Other Coping Strategies for Anxiety

Other responses to anxiety to which women resorted in pregnancy, ones which are perhaps less conducive to improved mental health, included isolating or silencing oneself, taking emotions out on children, and expressing fatalistic notions of resignation. These strategies were more commonly employed by women with limited enabling resources and agency such as Raabia, who described her husband’s response to her attempts to share feelings as follows: “He says, ‘Be patient and *Allah* will make things better.’ Then I feel tension. I keep silent. Who should I tell?” (M13). In contrast to the faith-based acceptance described earlier, such notions of resignation to one’s fate appeared as a rationale for some women to not access support, not seek care from a provider, or avoid addressing their anxieties. When asked how providers might address her anxieties about child delivery, Zara responded, “Doctors cannot do anything. It’s all in *Allah*’s hands. It will go away on its own” (M14). A few other mothers described isolating themselves and keeping silent as their best or only recourse for coping with the sources or symptoms of anxiety. This could include separating oneself (“I become silent and I go to my room (…) I just sit in my room silently” M13), weeping alone (“I get quiet, start weeping in my room” M15), or talking to oneself (“I take my mirror as my friend, that acts as my sister (…) a big mirror so I can talk in front of it” M11). Fatalism and silence were in some cases encouraged by the family members with whom women shared their problems, such as Raabia’s husband in the excerpt above. In another example, Erum’s parents gave her advice regarding her anxieties about conflict with her husband or in-laws if she gives birth to another daughter: “My parents say keep quiet. Now I am silent (…) My parents say everything is fine if you keep your mouth shut” (M1).

A few pregnant mothers described taking out emotions on their children, in some cases through physical abuse. Hawa described becoming easily irritated by her children’s behavior and resorting to beating them (“if children get into mischief, then I often beat them, I get angry very often” M9), whereas Raabia stated the following when asked why she beats her son: “When I feel worries, when I feel tension, and also when I feel anger (…) I have beaten him a lot” (M13). With corporal punishment for children being a normative behavior among mothers, these women described relying more heavily on beatings or physical abuse for either managing their children or as an outlet for other emotions, especially among those who described limited perceived agency or benefit in exercising alternative coping strategies. As a contrasting example, Basma sought relief from anxiety-causing fears of dying during childbirth by talking to her nine-year-old daughter about this possibility and placing on her the responsibility of caring for her siblings in her mother’s absence: “I sat down with my daughter and told her that if I die and your father gets married again, then look after your siblings” (M2).

## 4. Discussion

This is the first qualitative study to explore empowerment and the sociocultural experience of prenatal maternal anxiety in South Asia. Our focus is particularly relevant given the disproportionate burden of anxiety during pregnancy in Pakistan [13,14], the scarcity of research on prenatal maternal anxiety in LMICs [2,3], and insufficient attention to the relationship between women’s empowerment and pregnancy or childbirth in general [43]. This study contributes toward improving our theoretical understanding of the intersections between women’s empowerment and experiences of prenatal anxiety by demonstrating how maternal decision-making, levels of social support, and gender norms may interact as sources, mitigators, or exacerbators of anxiety during pregnancy as well as facets of the multi-dimensional construct of empowerment. Viewed through an empowerment lens as part of enabling resources and agency, these factors can be understood as enabling or constraining pregnant women’s use of coping strategies that would facilitate the achievement of improved mental health (Figure 1) [22]. 

Our findings suggest that barriers to gaining voice in pregnancy for Pakistani women include fears of upsetting her *susral* (husband’s family) and that such fears are significant subjective contributors to prenatal anxiety. Avenues for exercising self-advocacy to address these and other sources of pregnant women’s anxieties are largely limited to indirect means, though levels of pregnancy-related agency also depend on the area of decision-making, such as diet-related decisions versus access to prenatal care. Our findings are consistent with prior research demonstrating the importance of social relations (particularly control by husbands and interference by in-laws) in the experience of prenatal stress and CMDs, as well as in accessing prenatal care within the Pakistani context. For example, one study in Karachi both qualitatively and quantitatively identified social relations involving husbands and in-laws as a significant determinant of perinatal depression [44]. An integrated analysis of ethnographic and survey data in Punjab found interpersonal ties with the mother-in-law and husband to also be important in accessing antenatal care, though this effect was moderated by social class [45]. Moreover, the varying levels of agency across the areas of decision-making shown in our data are consistent with results from focus group research in rural Punjab, which found that food purchasing was the only area of family decision-making in which the Pakistani women reported playing a major role [46].

Our study also underscores the critical role of peer and family support as a protective factor against prenatal anxiety and as an enabling resource for maternal mental health more generally. The results of our qualitative investigation are consistent with quantitative studies that show low marital satisfaction and low social support to be significant psychosocial risk factors for CMD symptoms among pregnant women in various Asian populations [13,47,48,49,50]. A meta-ethnography synthesizing the results of five qualitative studies on perinatal mental illness also identified isolation from peer support to be an important theme across women’s experiences [51].

Gender norms emerged as another dimension of women’s social environment that is important in shaping experiences of prenatal anxiety. While prior studies of maternal mental health in Pakistan have focused on domestic abuse or intimate partner violence as risk factors for perinatal CMDs [52,53], a strength of our qualitative investigation is the rich elucidation of how subtler manifestations of gender inequality—unequal control of household finances, restrictions on women’s mobility, and expectations or preferences for a male child—intersect with anxiety and empowerment in the period of pregnancy. Moreover, by exploring gender norms from the perspective of pregnant women, our study extends the existing literature on prenatal maternal anxiety [3], yielding results that contextualize prior descriptions of patriarchal control over economic and health care decisions in South Asia [27,54]. For example, accompanied mobility in accessing prenatal health services was cited by some women in our study as evidence of strong social support and positive intra-household relationships rather than of circumscribed freedom of movement. Such accounts help illustrate the complex nature of female movement and complicate the dominant discourse on “travel alone” within the broader paradigm of women’s autonomy as conceptualized by Western feminists [55,56].

Similarly, our exploration of coping strategies for anxiety produced a multifaceted picture, including approaches that are ostensibly conducive to improved mental health and others that may have deleterious effects on social and mental well-being or serve as barriers to care seeking for anxiety. The role of faith-based coping mechanisms appeared mixed, with some women conveying fatalistic helplessness or hopelessness about their anxiety while others portrayed faith or religious practices as sources of acceptance, comfort, and even self-reliance. The long-established construct of fatalism in health behavior research is partly challenged by such nuances and apparent contradictions in the women’s firsthand accounts of how their responses to anxiety in pregnancy are influenced and informed by their faith. This supports previous research demonstrating the contestability of fatalism as used to explain the health behaviors of populations [57,58,59] and the need to critically examine the factors behind patients’ use of culturally acceptable idioms of fatalism [60,61].

### Study Limitations

A notable limitation of this study was its reliance on the secondary analysis of data collected for the primary purpose of informing the design of a preventive intervention targeting clinical or subclinical symptoms of anxiety in pregnancy for subsequent testing in a randomized trial [62]. As such, the original data collection and sampling procedures were designed to optimize the identification of cultural and familial risk factors for anxiety in pregnancy without the explicit consideration of pregnancy-related empowerment constructs. The semi-structured in-depth interviews were nonetheless based on open-ended questions to elicit narratives of individual experiences with stressors and coping strategies [63,64], thereby providing rich data with thick descriptions of the pregnant women’s sources of anxiety and social context. Additionally, care was taken to enhance the credibility of the secondary analysis through the use of two independent research team members for the open coding of initial transcripts (from which empowerment-related themes such as decision-making consistently emerged) as well as consultation with Pakistani colleagues to support the appropriate interpretations and descriptions of sociocultural factors. 

Second, several interview topics, such as pregnant women’s treatment by husbands and in-laws, were potentially sensitive. Despite our best efforts to conduct interviews in quiet private areas and assure participants of confidentiality, maltreatment or abuse may have been underreported by women. Still, for the interview setting, the health facility was preferable to women’s homes, where fears regarding the disclosure of negative experiences or neglect and abuse by family would be more acute, given the norms of having a family member such as a mother-in-law present for such encounters [65,66]. To minimize the extent to which stigma associated with psychiatric conditions prevented women from openly discussing experiences of anxiety, interviewers were trained to avoid terms such as “anxiety” or “disorder” and instead use terms like “tension”, “stress”, or “worry”. This was based on previous research experience showing that such terms are less stigmatizing, particularly in Pakistani settings and the local language [67,68]. This may have influenced study participants in ways that reduced reporting on how stigma against care seeking for mental health can itself be a source or exacerbator of anxiety or shape women’s coping strategies.

Thirdly, interviews were conducted at a single timepoint in participants’ pregnancies without subsequent follow-up. In the absence of multiple interviews, no temporal processes of empowerment and anxiety across pregnancy could be adequately explored or evaluated. As such, our study provides only a limited glimpse into the processes of decision-making and experiences of prenatal anxiety among participants. Despite the resulting inability to draw inferences from this data on temporal relationships or associations between facets of power during, before, or after pregnancy, our results still offer an opportunity to appreciate the interrelated and inseparable nature of agency, resources, and achievements within this sample of women’s narratives about their subjective experiences of anxiety in the prenatal period. This includes the tendency among study participants who described greater voice or social support to have also reported reliance on more health-conducive coping strategies for anxiety during pregnancy, a phenomenon that warrants further study and rigorous examination in future research.

Lastly, although depression is highly co-morbid with post-traumatic stress disorder (PTSD) and was an exclusion criterion [69], the absence of a specific assessment for PTSD or complex PTSD in the original screening protocol for participants was a limitation of this study, given the potential links between experiences or sources of anxiety and exposures or responses to trauma among pregnant women.

## 5. Conclusions

This study represents the first qualitative investigation of how perinatal anxiety in South Asia is potentially shaped by pregnancy-related empowerment based on descriptions of the sociocultural dimensions of anxiety from the perspectives of symptomatic expectant mothers. A key strength of this study is its application of constructs specific to pregnancy-related empowerment that capture not only women’s voice and participation in decision-making relevant to their health but also the connectedness women feel with family, peers, and caregivers in the context of pregnancy [23]. The study’s analytic approach thus facilitated an in-depth exploration of the unique features of prenatal anxiety in the South Asian context, especially those related to male gender preferences and patrilocal joint-family arrangements. We found that women’s voice in decisions impacting their health and pregnancy, the availability of enabling resources such as social support, and the extent to which empowerment is constrained by manifestations of gender norms in pregnancy are important factors shaping women’s sources of and coping strategies for prenatal anxiety. This study suggests that pregnancy-related empowerment can be useful for contextualizing the experience of prenatal maternal anxiety for women in South Asia, and a further examination of the intersections between empowerment and perinatal mental illness is warranted. This is of particular relevance in settings like South Asia where the high burden of CMDs in pregnancy exists within a context of rapid changes in the social and economic environment, including the available employment opportunities, level of education, and traditional gender roles for women [70].

While our study focused on perceived sources of anxiety from the perspective of pregnant women and was therefore limited to interviews with expectant mothers, future triangulation with husbands or other family members may help to more comprehensively capture the role of family dynamics, social support, or interpersonal conflict in influencing women’s mental health in pregnancy. Additionally, women’s empowerment itself still needs further conceptualization in future research. Importantly, measures of women’s agency may not only mediate the impact of psychosocial interventions on prenatal anxiety but also themselves be associated with women’s anxiety [21,43]. As a distinct psychosocial phenomenon, pregnancy-related empowerment does not necessarily mirror commonly measured economic or sociodemographic factors [71]. Nonetheless, these findings support the importance of researchers and practitioners paying greater attention to the relationships between women’s empowerment and mental health, especially in the critical vulnerable period of pregnancy.

## Figures and Tables

**Table 1:** Items of the Hospital Anxiety and Depression Scale (HADS) † subscale for anxiety [34].

Number	Item Text (in English)
1	I feel tense or “wound up.”
3	I get a sort of frightened feeling as if something awful is about to happen.
5	Worrying thoughts go through my mind.
7	I can sit at ease and feel relaxed. *
9	I get a sort of frightened feeling like butterflies in my stomach.
11	I feel restless, as if I have to be on the move.
13	I get a sudden feeling of panic.

Note: *—This item is reverse coded; †—From Zigmond & Snaith, 1983.

**Table 2:** Demographic Profile of Study Participants (n = 19).

Interviewee (Pseudonym)	Age (in years)	Number/Gender of Live Children	Marital Status	Length of Education	Family Structure	Other Notes
M1 (*Erum)*	24	1 (girl)	Married	10 years	Nuclear	
M2 (*Basma*)	28	3 (1 boy, 2 girls)	Married	12 years	Nuclear	
M3 (*Pashmina*)	21	0	Married	12 years	Joint	First pregnancy, recent death of mother-in-law
M4 (*Tasleem*)	30	2 (2 girls)	Married	12 years	Joint	One boy died in delivery
M5 (*Teena*)	26	0	Married	10 years	Joint	
M6 (*Sobia*)	33	0	Married	10 years	Nuclear	Two prior miscarriages
M7 (*Jumana)*	24	2 (1 boy, 1 girl)	Married	5 years	Joint	<2 yeargap with previous pregnancy
M8 *(Chaandni)*	18	0	Married	5 years	Joint	Second trimester, 1st pregnancy
M9 *(Hawa)*	24	2	Married	10 years	Joint	
M10 *(Khadija)*	24	2 (2 sons)	Married	5 years	Nuclear	One prior miscarriage and three infants who died post-delivery
M11 *(Deena)*	23	0	Married	14 years	Joint	
M12 *(Zulekha)*	24	0	Married	6 years	Joint	
M13 *(Raabia)*	37	1 (boy)	Married	10 years	Joint	Husband’s job abroad
M14 *(Zara)*	20	2	Married	5 years	Nuclear	
M15 *(Saaliha)*	27	1 (girl)	Married	16 years	Joint	Employed as tutor; husband’s job abroad
M16 *(Shafaq)*	32	0	Married	14 years	Joint	
M17 *(Taahira)*	21	0	Married	12 years	Joint	
M18 *(Zahirah)*	34	4	Married	14 years	Nuclear	
M19 *(Aaliya)*	25	0	Married	10 years	Joint	
